# Tryptophan Supplementation Enhances Intestinal Health by Improving Gut Barrier Function, Alleviating Inflammation, and Modulating Intestinal Microbiome in Lipopolysaccharide-Challenged Piglets

**DOI:** 10.3389/fmicb.2022.919431

**Published:** 2022-07-04

**Authors:** Guangmang Liu, Jiajia Lu, Weixiao Sun, Gang Jia, Hua Zhao, Xiaoling Chen, In Ho Kim, Ruinan Zhang, Jing Wang

**Affiliations:** ^1^Key Laboratory for Animal Disease-Resistance Nutrition, Ministry of Education, Ministry of Agriculture and Rural Affairs, Key Laboratory of Sichuan Province, Institute of Animal Nutrition, Sichuan Agricultural University, Chengdu, China; ^2^Department of Animal Resource and Science, Dankook University, Cheonan, South Korea; ^3^Maize Research Institute, Sichuan Agricultural University, Chengdu, China

**Keywords:** tryptophan, intestinal barrier function, microbiota, inflammation, piglets

## Abstract

Tryptophan (Trp) can modify the gut microbiota. However, there is no information about the effect of Trp on intestinal microbiota after lipopolysaccharide (LPS) challenge. This study aimed to investigate the effect of Trp on intestinal barrier function, inflammation, antioxidant status, and microbiota in LPS-challenged piglets. A total of 18 weaned castrated piglets were randomly divided into three treatments with 6 replicate per treatment, namely, (i) non-challenged control (CON); (ii) LPS-challenged control (LPS-CON); and (iii) LPS + 0.2% Trp (LPS-Trp). After feeding with control or 0.2% tryptophan-supplemented diets for 35 days, pigs were intraperitoneally injected with LPS (100 μg/kg body weight) or saline. At 4 h post-challenge, all pigs were slaughtered, and colonic samples were collected. The samples were analyzed for gut microbiota, fatty acids, antioxidant parameters, and the expression of mRNA and protein. The community bar chart showed that Trp supplementation to LPS-challenged pigs increased the relative abundance of *Anaerostipes* (*P* < 0.05) and tended to increase the relative abundance of *V9D2013_group* (*P* = 0.09), while decreased the relative abundance of *Corynebacterium* (*P* < 0.05) and *unclassified_c__Bacteroidia* (*P* < 0.01). Gas chromatography showed that Trp increased the concentrations of acetate, propionate, butyrate, and isovalerate in the colonic digesta (*P* < 0.05). Trp reduced the mRNA level of pro-inflammatory cytokines (*P* < 0.01), and increased mRNA level of aryl hydrocarbon receptor, cytochrome P450 (*CYP*) *1A1* and *CYP1B1* (*P* < 0.05). Correlation analysis results showed that acetate, propionate, and butyrate concentrations were positively correlated with mRNA level of *occludin* and *CYP1B1* (*P* < 0.05), and were negatively correlated with pro-inflammatory cytokines gene expression (*P* < 0.05). Isovalerate concentration was positively correlated with catalase activity (*P* < 0.05), and was negatively correlated with pro-inflammatory cytokines gene expression (*P* < 0.05). Furthermore, Trp enhanced the antioxidant activities (*P* < 0.01), and increased mRNA and protein expressions of claudin-1, occludin, and zonula occludens-1 (*P* < 0.01) after LPS challenge. These results suggest that Trp enhanced intestinal health by a modulated intestinal microbiota composition, improved the short chain fatty acids synthesis, reduced inflammation, increased antioxidant capacity, and improved intestinal barrier function.

## Introduction

Weaning is a critical period for the piglets’ growth. The physiological, nutritional, and environmental stress caused by problems such as intestinal barrier and inflammation leads to intestinal infections and performance reduction (Chen et al., 2018; Gao et al., 2019). Gut microbial dysbiosis is a major cause of intestinal infection and post-weaning diarrhea. There is a complex interaction between the intestinal flora and the host. Intestinal microorganisms produce a variety of metabolites (short-chain fatty acids [SCFAs], synthetic amino acids and vitamins), which not only affect digestion, absorption, and metabolism of nutrients, but also affect the growth of intestinal mucosa and the health growth of the host (Schwiertz et al., 2010; Ang and Ding, 2016; Ohira et al., 2017; Wang et al., 2018). The imbalance of gut microbiota structure leads to the disturbance of the body’s physiological function balance, colon cancer (Garrett, 2019), inflammatory bowel disease (Matsuoka and Kanai, 2015), intestinal stress syndrome (Lee and Bak, 2011), and other intestinal diseases. Gut microbial colonization has a significant effect on the development of innate and adaptive immune responses, and the homeostasis of intestinal barrier function (Nicholson et al., 2012). Dietary nutrients have a positive effect on intestinal homeostasis, host digestion, absorption, and immunity. In addition, it is necessary to use nutritional strategies in maintaining the health of the intestine. Amino acids are crucial for maintaining gut tissue integrity and the growth of microbiota (Liao, 2021).

The tryptophan (Trp) is regarded as the second-limiting amino acid in most corn–soybean diets of piglets (Mao et al., 2014), which must be supplied from the feed (Bravo et al., 2013). Previous studies have demonstrated that Trp supplementation increases feed intake (Trevisi et al., 2009), growth (Shen et al., 2012), gut integrity (Koopmans et al., 2006), and antioxidant status (Jacobitz et al., 2021; Liu et al., 2022). In addition, dietary Trp enhances intestinal cell protein turnover, tight junction protein expression (Tossou et al., 2016), and microbiota diversity (Dai et al., 2015), thereby improving intestinal barrier function. Moreover, Trp supplementation attenuates intestinal inflammation-induced increases of intestinal permeability, and the pro-inflammatory cytokine gene expression in pigs (Kim et al., 2010; Liu et al., 2022). Furthermore, being used for protein synthesis *in vivo*, Trp can also be catabolized through various pathways, such as kynurenine, serotonin, and gut microbiota metabolic pathways (Saraf et al., 2017; Agus et al., 2018). A growing number of studies showed that the metabolism of Trp involved in many diseases, such as inflammatory bowel disease, neurodegenerative diseases, and psychiatric disorders (Bosi et al., 2020). Trp can be directly catabolized by bacteria in the intestinal, forming indole, and its derivatives, which are involved in intestinal permeability, inflammation regulation, and host immunity (Lamas et al., 2016; Gao et al., 2018). Some Trp metabolites are regarded as ligands for aryl hydrocarbon receptor (AhR), improve local interleukin production and immunity. In addition, AhR is crucial for the renewal of intestinal epithelial cells and the integrity of the intestinal mucosal barrier (Nikolaus et al., 2017). Moreover, cytochrome P450 (CYP)1A1 acts as a direct transcriptional target of AhR constituting a feedback loop of AhR signaling (Schiering et al., 2017). 0.5% Trp supplementation inhibits colitis symptoms and the secretion of pro-inflammatory cytokines in mice by activating AhR (Islam et al., 2017). Trp metabolites increase the protein expression of zonula occluden (ZO)-1 and occludin (Liang et al., 2018b). However, there is no information about the effect of Trp on intestinal microbiota after lipopolysaccharide (LPS) challenge. LPS irritates the intestine, causing mucosal injury, metabolic disorder, and bacterial translocation, it has been used to mimic features of endotoxin-induced acute intestinal injury (Xu et al., 2018). In this study, LPS was injected to establish an intestinal inflammation model, and our objective was to test the hypothesis of whether Trp can improve gut barrier function, alleviate inflammation, and modulate intestinal microbiome in LPS-challenged piglets.

## Materials and Methods

### Experimental Design and Animals

The experimental protocol used in this study was approved by Sichuan Agricultural University Animal Care and Use Committee (SICAU-2021-08). A total of 18 castrated piglets (Duroc × Yorkshire × Landrace; weaned at 24 ± 1 days of age) were randomly distributed to three treatments. Each group has six replicates (one pig per replicate and one pen per pig). According to a previous study (Liu et al., 2016), the room temperature and relative humidity were adjusted to 30°C and 50–60%, respectively. The piglets were allowed to access clean water during the whole experiment. The basal diet (Table 1) was formulated based on the National Research Council (Southern and Adeola, 2012) requirements for all nutrients.

**TABLE 1:** Ingredient composition of experimental diets (%, as-fed basis).

Ingredient	Content (%)	
	
	7–11 kg	11–25 kg
Corn meal	27.37	30.6
Extruded corn	30.84	32.00
Soybean oil	2.50	1.40
Glucose	2.00	2.00
Whey powder	5.00	5.00
Dehulled Soybean meal (46% CP)	13.24	13.04
Soybean protein concentrate	5.00	5.00
Extruded soybean	7.00	5.00
Fish meal (67% CP)	3.00	2.50
L-Lysine-HCl (78.8%)	0.52	0.44
DL-Methionine (99%)	0.11	0.08
L-Threonine (98.5%)	0.20	0.15
L-Tryptophan (98%)	0.03	0.01
L-Alanine (99%)	0.46	0.32
Choline chloride (50%)	0.15	0.15
Limestone	0.68	0.41
Monocalcium phosphate	1.35	1.35
NaCl	0.25	0.25
Vitamin premix^1^	0.05	0.05
Mineral premix^2^	0.25	0.25
Total	100.00	100.00

**Nutrient level^3^**	**Content (%)**	

Digestible energy, Mcal/kg	3.55	3.49
Crude protein, %	19.72	18.65
Calcium, %	0.80	0.68
Total phosphorus, %	0.66	0.64
Available phosphorus, %	0.48	0.46
SID-Lysine, %	1.36	1.24
SID-Methionine, %	0.40	0.36
SID-Threonine, %	0.80	0.73
SID-Tryptophan, %	0.23	0.20

*^1^The vitamin premix provides the following per kilogram of diet: VA 15000 IU; VD_3_ 5000 IU; VE 40 IU; VK_3_ 5 mg; VB_1_ 5 mg; VB_2_ 12.5 mg; VB_6_ 6 mg; VB_12_ 600 μg; D-pantothenic acid 25 mg; nicotinic acid 50 mg; folic acid 2.5 mg; biotin 2.5 mg.*

*^2^The mineral premix provides the following per kilogram of diet: copper (CuSO_4_⋅5H_2_O) 6 mg; iron (FeSO_4_⋅H_2_O) 100 mg; zinc (ZnSO_4_⋅H_2_O) 100 mg; manganese (MnSO_4_⋅H_2_O) 4 mg; iodine (KI) 0.14 mg; selenium (Na_2_SeO_3_) 0.3 mg.*

*^3^Nutrient levels are calculated values.*

The experiment was carried out for a total of 35 days, and the experiment was divided into two stages according to the feeding weight: (1) 7–11 kg; (2) 11–25 kg. The experiment included three treatments: (1) non-challenged treatment (CON, the pigs were administered a control diet and received a 0.9% NaCl injection); (2) LPS-challenged treatment (LPS-CON, the pigs were given the same diet as the control group and were treated with *Escherichia coli* [*E. coli*] LPS); and (3) LPS-challenged + 0.2% tryptophan (Trp) treatment (LPS-Trp, the pigs were given with 0.2% Trp [CJ International Trading Co., Ltd.] diet and were treated with *E. coli* LPS [*E. coli* serotype 055: B5; Sigma Chemical Inc., St. Louis, MO, United States]). The Trp concentration was selected according to the previous research (Liang et al., 2018a). On the 35th day of the treatment, the challenged groups were intraperitoneally injected with *E. coli* LPS at 100 μg/kg body weight, and the unchallenged group was injected with the same amount of 0.9% saline. The LPS dose was selected in accordance with the previous research (Pi et al., 2014). At the end of the experimental trial, corresponding to day 35, all piglets fasted for 4 h before being slaughtered to avoid the potential effects of changes in feed intake in the intestine.

### Intestinal Sample Collection

On the 35th day of the treatment, 4 h after all pigs were intraperitoneally injected with *E. coli* LPS solution or sterile saline, 6 piglets from each treatment (one pig per pen) were electrocuted. After all pigs were sacrificed, each intestinal segment was ligated and separated, the colon was washed with saline, and a 2 cm middle segment of the colon was cut, each sample was collected at the same location. The colonic mucosa was scraped with glass slides for the determination of some immune indexes, some colon tissue samples were taken into sterilized EP tubes, quick-frozen in liquid nitrogen, and stored at −80°C for determination of antioxidants and genes. Colon contents were collected and aliquoted into sterile EP tubes for gut microbiome and volatile fatty acid determination.

### Short-Chain Fatty Acids Analysis

In each digesta sample, the main concentrations of SCFAs was separated and quantified by using a gas chromatograph (CP3800, Varian) with capillary column 30 m × 0.53 mm × 1 μm film thickness (HP-FFAP) and flame ionization detector, 250°C according to previous study (Zamora-Gasga et al., 2014). Standard samples (e.g., acetate, propionate, butyrate, isovalerate, isobutyrate, and valerate, Supelco, Sigma-Aldrich Trading Co., Ltd., Shanghai, China) were used. Briefly, 0.7 g of sample was collected in a centrifuge tube, and was mixed with ultrapure water (1.5 ml) for 30 min and centrifuged (1,000 g, 15 min). The supernatant (1 ml) was added 0.2 mL of 25% (w/v) metaphosphate solution (Tianjin Kemiu Chemical Reagent Co., Ltd., Tianjing, China) and 23.3 μl of 210 mmol/l crotonic acid solution (Sigma-Aldrich Trading Co., Ltd., Shanghai, China), and the mixture was incubated for 30 min and then centrifuged at 8,000 g for 10 min. The supernatant (0.3 ml) was mixed with 0.9 ml of chromatographic methanol (Thermo Fisher Scientic Inc., Waltham, MA, United States) at 8,000 g for 5 min. One milliliter of the supernatant was subjected to capillary gas chromatography (CP3800, Varian).

### 16S rRNA Analysis of Colonic Digesta-Associated Microbiota

The method to extract the genomic DNA using FastDNA^®^ Spin Kit (MP Biomedicals, Irvine, CA, United States) was according to previous study (Layton et al., 2006). Briefly, 0.5 g sample was added with 978 μl sodium phosphate buffer and 122 μl MT buffer, and homogenized for 40 s (FastPrep-24 5G, MP Biomedicals, United States) and centrifuged (14,000 g, 5–10 min). 250 μl of PPS was added to the supernatant mixed and centrifuged (14,000 g, 5 min). About 600 μl of the mixture was placed in a SPIN™ filter and centrifuged (14,000 x g, 1 min). Finally, 500 μl of prepared SEWS-M was added, centrifuged at room temperature (14,000 rpm, 2 min), discarded the SPIN™ Filter. Total DNA was obtained. After genomic DNA extraction, the extracted genomic DNA was determined by 1% agarose gel electrophoresis. The ABI Gene Amp 9700 PCR Thermocycle Instrument (Applied Biosystems, Inc., Carlsbad, CA, United States) was applied to amplify DNA. Next, all samples were carried out according to formal experimental conditions. Each sample was repeated 3 times. PCR products from the same sample were blended and identified on a 2% agarose gel, then recovered by gel cutting with an AxyPrepDNA Gel Recovery kit (Axygen, Union City, CA, United States) and detected using a QuantiFluor-ST Handheld Fluorometer with UV/Blue Channels (Promega, Madison, WI, United States). The PCR products were sequenced on Miseq after concentration normalization (Illumina Inc., San Diego, CA, United States).

### Bioinformatics Analysis

The Paired-end reads generated by Miseq sequencing were first stitched together according to overlap relationships, and the sequence quality was checked and filtered. Usearch (version 7.0.1090^1^) was used to accomplish operational taxonomic units (OTU) clustering analysis (similarity cutoff rate of 97%). For classification analysis (confidence threshold 70%), each 16S rRNA gene sequence was compared to the Silva 16S rRNA database (version 138^2^) using the ribosomal database project classifier (version 11.5^3^). Alpha diversity index analysis on account of OTU clustering data from Mothur (version 1.30.2^4^). Qiime (version 1.9.1^5^) was used to conduct principal coordinate analysis (PCoA) on account of bray-curtis distance, and subsequently ANOSIM analysis on account of bray-curtis distance to evaluate clear differences among samples.

### Measurement of the Antioxidant Parameters

Antioxidant-related parameters were evaluated employing commecial kits (Jiancheng Bioengineering Institute, Nanjing, China) to determine the antioxidant activity of the colonic mucosa. Briefly, the catalase (CAT) activity, the malondialdehyde (MDA) content, the total superoxide dismutase (T-SOD), and the glutathione peroxidase (GSH-Px) activities were evaluated in conformity with earlier studies (Cao et al., 2016).

### Real-Time PCR Analysis

The real-time PCR analysis methods were in accordance with a previous experiment (Cao et al., 2017). Briefly, samples were extracted with Trizol (Takara, Dalian, China), and total RNA was dissolved in diethyl pyrocarbonate (Beyotime Biotechnology, Shanghai, China). The concentration and purity of total RNA were spectrophotometrically measured at OD260 and OD280 according to a previous study (Fang et al., 2017). Subsequently, the total RNA from each colon sample was utilized to transcribe into cDNA with the Prime Script™ RT reagent kit, as well as gDNA Eraser (Takara, Dalian, China). Our study utilized Primer Express Software (version 3.0; Applied Biosystems, Foster City, CA, United States) to design gene-specific primers, which were produced by Takara Biotechnology Company (Takara, Dalian, China). The forward and reverse primers of the genes were shown in Supplementary Table 1. The reaction system and PCR procedure was in conformity to our previous study (Fang et al., 2017). Samples was carried out on Real-Time PCR System (ABI 7900HT, Applied Biosystems), and the total volume of system is ten microliters [2 μl of cDNA, 2 μl of ddH_2_O water, 0.5 μl each of both reverse and forward primer and 5 μl SYBR^®^ Premix Ex Taq _II (TaKaRa, Dalian, China)]. The reaction condition was as follows: 41 cycles of 95°C for 30 s, followed by 95°C for 10 s and 58°C for 35 s. A housekeeping gene β-actin (ACTB) was utilized for data normalization. Relative mRNA expression was calculated in conformity to the 2^–ΔΔ^*^ct^* method.

### Western Blot

The Western blotting analysis was tested on the basis of the steps by Chen et al. (2016). In brief, 0.1 g of colonic mucosal tissue in 900 μL RIPA lysis buffer with 1% phenylmethanesulfonyl fluoride and 2% phosphatase inhibitor cocktail A, 50X (Beyotime, Shanghai, China) was homogenized at 4°C. The pulverized tissues were centrifuged (13,000 g, 15 min) at 4°C, and the supernatant was collected for western blot analysis. The protein concentration of sample was tested by using the Enhanced BCA Protein Assay kit (Beyotime, Shanghai, China) to normalize. After normalization, 5 × loading buffer in a ratio of 1:4 was added and the proteins were boiled for 10 min to denature. The total proteins in the colonic mucosa were isolated and transferred to polyvinylidene fluoride membranes using sodium dodecyl sulfate polyacrylamide gel electrophoresis (Millipore, Eschborn, Germany). The running buffer is prepared by dissolving 3.03 g Tris base, 14.4 g glycine and 1 g SDS in double-distilled water to 1,000 mL. The electrophoresis process was 80 V constant pressure (30 min), and then changed to 120 V constant pressure (90 min), until the bromophenol blue moved to about 1 cm from the bottom of the gel and stopped. The total proteins in the colonic mucosa were isolated and transferred to polyvinylidene fluoride membranes using sodium dodecyl sulfate polyacrylamide gel electrophoresis (Millipore, Eschborn, Germany). The transfer buffer was prepared by dissolving 3.03 g Tris base, 14.4 g glycine and 200 mL methanol in double-distilled water to 1,000 mL. The transfer process was performed at a constant voltage of 100 V. Depending on protein molecular weight, claudin-1 was transferred for 32 min, occludin were transferred for 67 min and ZO-1 was transferred for 225 min, and β-actin was transferred for 52 min. After transfer, the membrane was washed twice with 1 × TBST for 5 min each at room temperature, pourd out 1 × TBST, and then dried it. The membrane was blocked with 5 ml 1 × TBST buffer containing 0.25 g fat-free milk at room temperature for 1 h. The blocking solution was discarded after blocking. The membrane was washed three times with 1 × TBST for 5 min each at room temperature, pourd out with 1 × TBST, and then dried it. The membrane was incubated with the primary antibody at room temperature for 60 min and overnight at 4°C. After overnight, the membrane was washed three times with 1 × TBST for 10 min each. The antibodies were used including ZO-1, occludin, and claudin-1 (1:1,000, Proteintech Group, Inc., Wuhan, China). Finally, the membrane was incubated with the rabbit second antibody at room temperature for 90 min. The target bands were visualized through a high-sensitivity multi-function imaging system (ChemiDoc™, Bio-Rad). Enhanced chemiluminescence was utilized to display the clarity western signals (Beyotime, Shanghai, China). Afterward, the intensity of the bands was determined using Image Lab software (version 6.1, Bio-Rad, Berkeley, CA, United States).

## Statistical Analysis

All data were evaluated with the independent-samples *t*-test using the SPSS (version 26, IBM, Chicago, IL, United States). A Shapiro–Wilk test was used as a test of normality. Levene’s test was employed to examine homogeneity of variances. All results were expressed as the mean ± standard error. In addition, we normalized the data to equalize the OTU sequence. Species were selected for correlation network graph analysis based on pearson correlation coefficient (Majorbio Bio-pharm Technology Co., Ltd, Shanghai, China). *P* < 0.05 was deemed statistically significant. *P*-values between 0.05 and 0.10 were used to identify the trends.

## Results

### Diversity of the Microbiome

**FIGURE 1:**
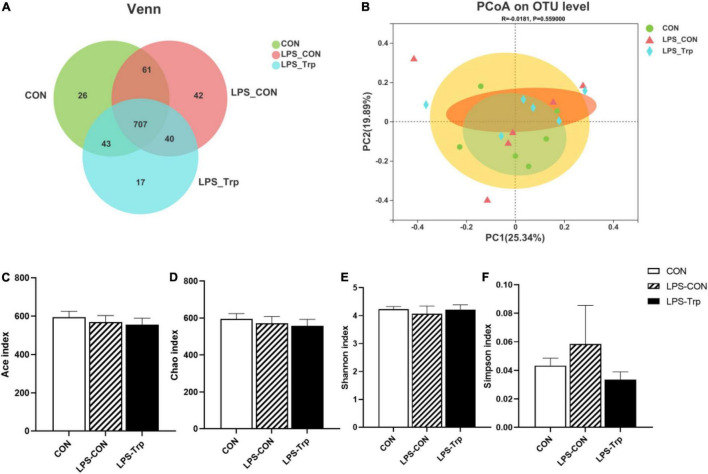
The effects of tryptophan supplementation on the diversity of colonic digesta in lipopolysaccharide-challenged piglets. CON, control group; LPS-CON, piglets challenged with lipopolysaccharide; LPS-Trp, piglets fed with tryptophan and challenged with lipopolysaccharide. **(A)** Venn diagram of core operational taxonomic units. **(B)** Principal coordinate analysis on colonic microbiota, significant differences were determined using ANOSIM analysis. **(C)** Ace index of colonic mucosa microbiota. **(D)** Chao index of colonic mucosa microbiota. **(E)** Shannon index of colonic mucosa microbiota. **(F)** Simpson index of colonic mucosa microbiota. Values are shown as mean ± standard error (*n* = 6).

In our experiment, we obtained a total of 1159521477743542 bases optimized sequences, with an average of 412 bases per sample. The OTUs were clustered with 97% similarity. The CON group, LPS-CON group, and LPS-Trp group each comprised 837, 850, and 807 core OTUs, respectively, whereas 707 core OTUs were common among all the three groups (Figure 1A). The three groups did not differ significantly in terms of PCoA diversity on the basis of bray-curtis distance (Figure 1B). The α diversity of gut microbiota was shown in Figures 1C–F. The Trp did not affect the shannon, simpson, ace, and chao indexes.

### Bacterial Abundance in the Colon

**FIGURE 2:**
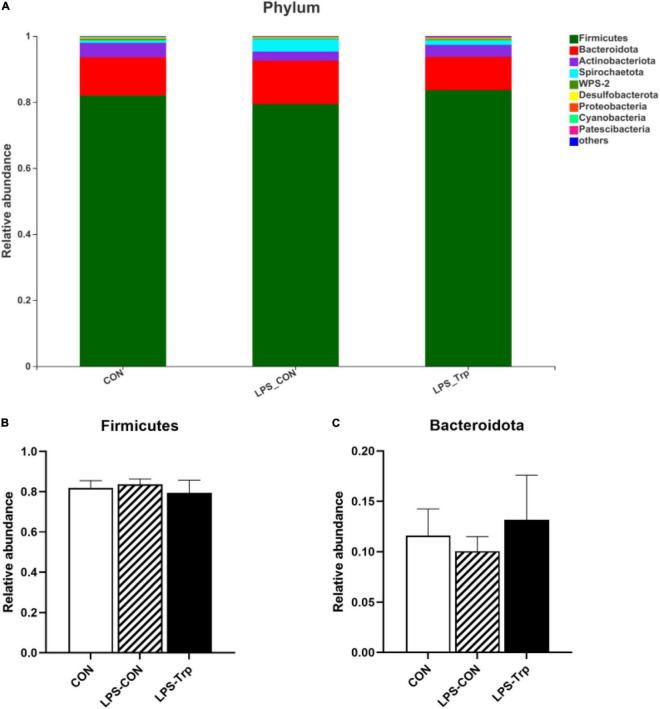
At the phylum level, the effects of tryptophan supplementation on the relative abundance of colonic digesta. CON, control group; LPS-CON, piglets challenged with lipopolysaccharide; LPS-Trp, piglets fed with tryptophan and challenged with lipopolysaccharide. **(A)** The graph showed the level composition of phylum. **(B)** The change of Firmicutes. **(C)** The change of Bacteroidota. Values are shown as mean ± standard error (*n* = 6).

The digesta microbiota composition was shown in Figure 2. At the phylum level, we identified two predominant phyla (e.g., Firmicutes and Bacteroidetes). Firmicutes accounted for 81.99, 83.67, and 79.40% in the CON, LPS-CON, and LPS-Trp treatments, respectively. Bacteroidetes accounted for 11.59, 10.04, and 13.16% in the CON group, LPS-CON group, and LPS-Trp group, respectively (Figure 2A). The abundances of Firmicutes and Bacteroidetes were shown in Figures 2B,C.

**FIGURE 3:**
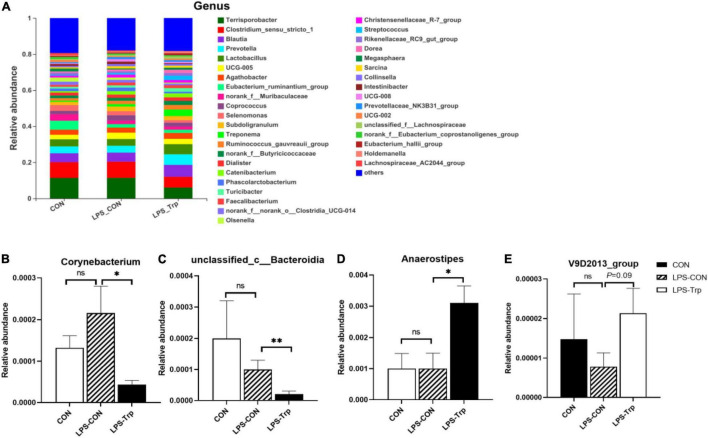
At the genus level, the effects of tryptophan supplementation on the relative abundance of colonic digesta. CON, control group; LPS-CON, piglets challenged with lipopolysaccharide; LPS-Trp, piglets fed with tryptophan and challenged with lipopolysaccharide. **(A)** The graph showed the level composition of genus. **(B)** The change of Corynebacterium. **(C)** The change of unclassified_c__Bacteroidia. **(D)** The change of Anaerostipes. **(E)** The change of V9D2013_group. Values are shown as mean ± standard error (*n* = 6). 0.05 ≤ *P* < 0.10 was considered as a tendency. *P < 0.05; **P < 0.01. “ns” was considered as not significant.

At the genus level, the genera (at least one treatment group) with relative abundances greater than 0.1% were shown in Figure 3. *Terrisporobacter* (CON group: 11.35%, LPS-CON group: 11.38%, LPS-Trp group: 6%), *Clostridium_sensu_stricto_1* (CON group: 8.74%, LPS-CON group: 8.96%, LPS-Trp group: 5.99%), *Blautia*, *Lactobacillus*, and *Prevotella* were the dominant genera (Figure 3A).

According to statistical analysis, compared with the LPS-CON group, the relative abundances of *Corynebacterium* (*P* < 0.05, Figure 3B) and *unclassified_c__Bacteroidia* (*P* < 0.01, Figure 3C) in the LPS-Trp group were significantly decreased. Moreover, the relative abundances of *Anaerostipes* was significantly increased (*P* < 0.05, Figure 3D), and the relative abundance of *V9D2013_group* tended to increase (*P* = 0.09, Figure 3E) in the LPS-Trp group.

### Short-Chain Fatty Acids Concentrations in Colonic Digesta

**FIGURE 4:**
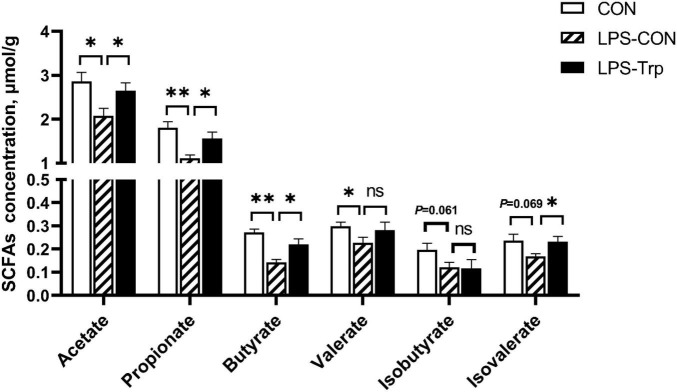
The effects of tryptophan supplementation on SCFAs concentrations in colon of lipopolysaccharide-challenged piglets. SCFAs, short-chain fatty acids; CON, control group; LPS-CON, piglets challenged with lipopolysaccharide; LPS-Trp, piglets fed with tryptophan and challenged with lipopolysaccharide. Values are shown as mean ± standard error (*n* = 6). 0.05 ≤ *P* < 0.10 was considered as a tendency. *P < 0.05; **P < 0.01. “ns” was considered as not significant.

The SCFAs concentrations are shown in Figure 4. Relative to the CON group, the LPS challenge significantly decreased the concentrations of acetate, valerate (*P* < 0.05), butyrate, propionate (*P* < 0.01), and the concentrations of isovalerate (*P* = 0.069) and isobutyrate (*P* = 0.061) tended to decrease. Compared with the LPS-CON group, the LPS-Trp group had higher acetate, propionate, butyrate, and isovalerate concentrations (*P* < 0.05) in colonic digesta, and no significant change was observed in the concentrations of valerate and isobutyrate.

### mRNA Expression of Colonic Mucosa

**FIGURE 5:**
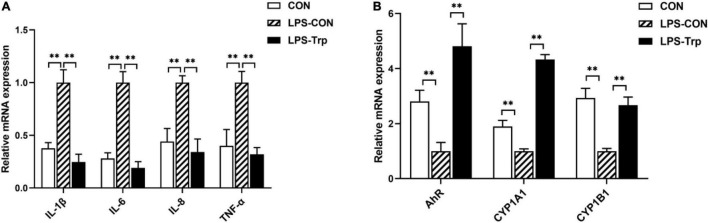
**(A)** The effects of tryptophan supplementation on the expression of colonic pro-inflammatory cytokines in the piglets. **(B)** The effects of tryptophan supplementation on the expression of colonic mucosa-related genes in the piglets. IL-1β, interleukin-1β; IL-6, interleukin-6; IL-8, interleukin-8; TNF-α, tumor necrosis factor-α; AhR, aryl hydrocarbon receptor; CYP1A1, cytochrome P4501A1; CYP1B1, cytochrome P4501B1; CON, control group; LPS-CON, piglets challenged with lipopolysaccharide; LPS-Trp, piglets fed with tryptophan and challenged with lipopolysaccharide. Values are shown as mean ± standard error (*n* = 6). **P < 0.01.

The mRNA expressions of colonic pro-inflammatory cytokines were shown in Figure 5A. Relative to the CON group, the LPS-CON group had significantly higher mRNA expressions of *IL-1*β, *IL-6*, *IL-8*, and *TNF*-α (*P* < 0.01). The LPS-Trp group had significantly lower mRNA expressions of *IL-1*β, *IL-6*, *IL-8*, and *TNF*-α (*P* < 0.01) than the LPS-CON group. The mRNA expressions of colonic mucosa were shown in Figure 5B. Relative to CON group, the LPS-CON group had significantly lower *AhR*, *CYP1A1*, and *CYP1B1* (*P* < 0.01) mRNA expressions. Compared with the LPS-CON group, the LPS-Trp group had significantly higher *AhR*, *CYP1A1*, and *CYP1B1* (*P* < 0.01) mRNA expressions.

### Antioxidant Indicators in the Colon

**FIGURE 6:**
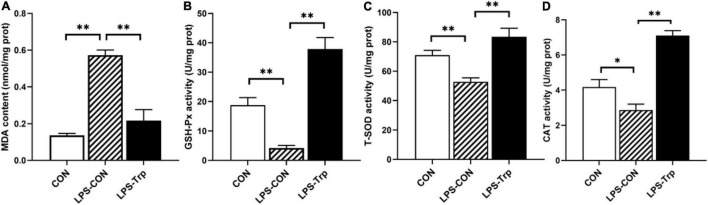
The effects of tryptophan supplementation on antioxidant capacity in colonic mucosa of piglets. MDA, malondialdehyde; GSH-Px, glutathione peroxidase; T-SOD, total superoxide dismutase; CAT, catalase; CON, control group; LPS-CON, piglets challenged with lipopolysaccharide; LPS-Trp, piglets fed with tryptophan and challenged with lipopolysaccharide. **(A)** The content of MDA. **(B)** The activity of GSH-Px. **(C)** The activity of T-SOD. **(D)** The activity of CAT. Values are shown as mean ± standard error (*n* = 6). 0.05 ≤ *P* < 0.10 was considered as a tendency. *P < 0.05; **P < 0.01.

The antioxidant indicators were shown in Figure 6. Relative to the CON group, the LPS challenge significantly decreased the activities of GSH-Px, T-SOD (*P* < 0.01) and CAT (*P* < 0.05), and significantly increased the content of MDA (*P* < 0.01). In addition, compared with the LPS-CON group, Trp supplementation significantly increased the activities of GSH-Px, T-SOD, and CAT (*P* < 0.01), and significantly declined the content of MDA (*P* < 0.01).

### Tight Junction Gene and Protein Expression Levels in Colonic Mucosa

**FIGURE 7:**
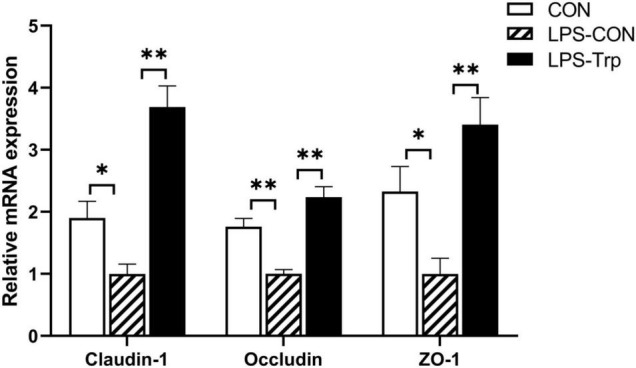
The effects of tryptophan supplementation on the mRNA expression of tight junction protein. ZO-1, zonula occludens-1; CON, control group; LPS-CON, piglets challenged with lipopolysaccharide; LPS-Trp, piglets fed with tryptophan and challenged with lipopolysaccharide. Values are shown as mean ± standard error (*n* = 6). *P < 0.05; **P < 0.01.

**FIGURE 8:**
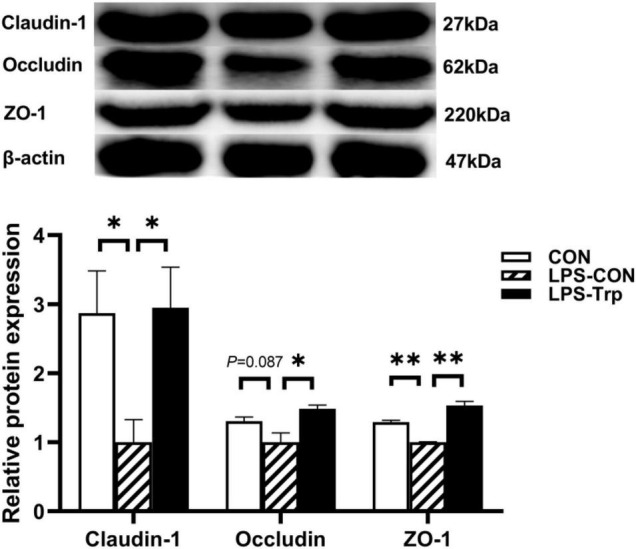
The effects of tryptophan supplementation on the ratios of claudin-1/β-actin, occludin/β-actin, and ZO-1/β-actin of weaning piglets after lipopolysaccharide challenge. ZO-1, zonula occludens-1; CON, control group; LPS-CON, piglets challenged with lipopolysaccharide; LPS-Trp, piglets fed with tryptophan and challenged with lipopolysaccharide. The bands shown are the representative western blot images of claudin-1 and occludin. Values are shown as mean ± standard error (*n* = 4). 0.05 ≤ *P* < 0.10 was considered as a tendency. *P < 0.05; **P < 0.01.

The effect of Trp supplementation on the mRNA expression of tight junction protein was shown in Figure 7. Relative to CON group, the LPS-CON group had significantly lower *claudin-1* (*P* < 0.01), *occludin* (*P* < 0.05), and *ZO-1* (*P* < 0.01) mRNA expression. Compared with the LPS-CON group, the LPS-Trp group had significantly higher *claudin-1*, *occludin*, and *ZO-1* (*P* < 0.01) mRNA expression. The effect of Trp supplementation on the expressions of tight junction protein was shown in Figure 8. Relative to CON pigs, the LPS-CON pigs had lower ratios of claudin-1/β-actin (*P* < 0.05), occludin/β-actin (*P* = 0.087), and ZO-1/β-actin (*P* < 0.01). Relative to LPS-CON pigs, the LPS-Trp pigs had higher ratios of claudin-1/β-actin, occludin/β-actin (*P* < 0.05), and ZO-1/β-actin (*P* < 0.01).

### Correlation Analysis Between the Gut Microbiota Metabolites and Inflammation, Antioxidant, Barrier-Relative Parameters in Piglets

**FIGURE 9:**
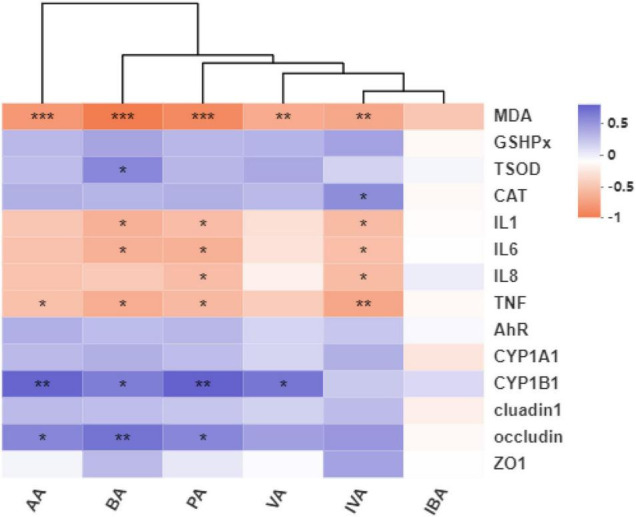
Heat maps of the pearson correlation coefficient and significant tests between the gut microbial metabolite SCFAs and antioxidant capacity, expressions of colonic mucosa-related genes. AA, acetate; BA, butyrate; PA, propionate; VA, valerate; IVA, isovalerate; IBA, isobutyrate; MDA, malondialdehyde; GSHPx, glutathione peroxidase; TSOD, total superoxide dismutase; CAT, catalase; IL1, interleukin-1β; IL6, interleukin-6; IL8, interleukin-8; TNF-α, tumor necrosis factor-α; AhR, aryl hydrocarbon receptor; CYP1A1, cytochrome P4501A1; CYP1B1, cytochrome P4501B1; ZO1, zonula occludens-1; CON, control group; LPS-CON, piglets challenged with lipopolysaccharide; LPS-Trp, piglets fed with tryptophan and challenged with lipopolysaccharide. *R* values were shown in different colors in the figure. The color range of different *R* values was shown in the legend on the right. Values are shown as mean ± standard error (*n* = 6). *P < 0.05; **P < 0.01; ***P < 0.001.

The heat maps of the Pearson correlation coefficient between the gut microbial metabolite SCFAs and antioxidant capacity and the expressions of colonic mucosa-related genes were shown in Figure 9. Acetate concentration was positively correlated with mRNA level of *occludin* (*r* = +0.054, *P* = 0.033) and *CYP1B1* (*r* = +0.669, *P* = 0.002), and was negatively correlated with MDA content (*r* = −0.716, *P* = 0.001) and *TNF*-α gene expression (*r* = −0.475, *P* = 0.047). Propionate concentration was positively correlated with mRNA level of *occludin* (*r* = +0.502, *P* = 0.034) and *CYP1B1* (*r* = +0.682, *P* = 0.002), and was negatively correlated with MDA content (*r* = −0.805, *P* = 0.000), and with mRNA level of *IL-1*β (*r* = −0.500, *P* = 0.035), *IL-6* (*r* = −0.566, *P* = 0.014), *IL-8* (*r* = −0.494, *P* = 0.037), and *TNF*-α (*r* = −0.522, *P* = 0.026). Butyrate concentration was positively correlated with mRNA level of *occludin* (*r* = +0.593, *P* = 0.010) and *CYP1B1* (*r* = +0.554, *P* = 0.020), and with T-SOD activity (*r* = +0.499, *P* = 0.035), and was negatively correlated with MDA content (*r* = −0.880, *P* = 0.000), and with mRNA level of *IL-1*β (*r* = −0.567, *P* = 0.014), *IL-6* (*r* = −0.569, *P* = 0.014), and *TNF*-α (*r* = −0.583, *P* = 0.011). Isovalerate concentration was positively correlated with CAT activity (*r* = +0.469, *P* = 0.050), and was negatively correlated with MDA content (*r* = −0.620, *P* = 0.006), and with mRNA level of *TNF*-α (*r* = −0.627, *P* = 0.005), *IL-1*β (*r* = −0.502, *P* = 0.034), *IL-6* (*r* = −0.486, *P* = 0.041), and *IL-8* (*r* = −0.504, *P* = 0.033).

## Discussion

Complex and various microbial community colonization is crucial in maintaining innate and adaptive immune responses and intestinal health (Nicholson et al., 2012; Gardiner et al., 2020). Diets regulate the microbial composition and diversity (Zhang et al., 2018). Trp regulates the composition and diversity of cecal microbia (Liang et al., 2018b). However, how the intestinal microbiota is regulated by Trp after LPS challenge remains unknown. The colon is the intestinal segment with the most active microbial metabolic activity (Sommer and Bäckhed, 2016). Thus, selecting the colon was emphasized in this study.

Colon microbiota was extracted to investigate the role of Trp on intestinal microbiota. At the phylum level in this study, we found that the majority of the microbiota composition was Firmicutes and Bacteroidota. Firmicutes produce SCFAs modulating intestinal homeostasis (Ellekilde et al., 2014; Louis and Flint, 2017). At the genus level, we found that the majority of the microbiota composition was *Terrisporobacter*, followed by *Clostridium_sensu_stricto_1*, *Blautia*, *Prevotella*, and *Lactobacillus*. Compared with the LPS-CON group, Trp supplementation decreased the proportion of *Terrisporobacter* and *Clostridium_sensu_stricto_1* abundance, as well as increased the proportion of *Blautia*, *Prevotella* and *Lactobacillus* abundances. *Terrisporobacter* is regarded as a member of the *Peptostreptococcaceae* family. The increased abundance of *Terrisporobacter* can induce oxidative stress and inflammation in the host (Cai et al., 2019). This is in agreement with our study that Trp supplementation to the LPS-challenged pigs decreased the proportion of *Terrisporobacter* abundance. *Blautia* occurs widely in the feces and intestines of mammals, as a genus of the *Lachnospiraceae* family, which modulates inflammation, metabolic disorders, and against specific microorganisms (Liu et al., 2021). Consistent with our finding, Trp supplementation to the LPS-challenged pigs increased the proportion of *Blautia* abundance. *Prevotella* belongs to *Bacteroidetes*, which produce SCFAs regulating intestinal homeostasis (Atarashi et al., 2011; Ramakrishna, 2013; Gonçalves et al., 2018). In addition, this is in agreement with our study that Trp supplementation to the LPS-challenged pigs increased the proportion of *Prevotella* abundance. *Lactobacillus* is crucial for regulating LPS-induced intestinal damage in piglets (Sugawara et al., 2020). The experiment showed that in the LPS-induced colitis assay, the cell wall content of *Lactobacillus* reduces immune inflammation and enhances antioxidant defense to prevent induced colitis in mice (Chorawala et al., 2021). Moreover, Trp supplementation to the LPS-challenged pigs increased the proportion of *Lactobacillus* abundance. Trp promotes the beneficial bacteria in the intestinal flora and inhibits the pathogenic bacteria (Krishnan et al., 2018; Liang et al., 2018b). This is crucial for the host’s intestinal health and systemic homeostasis (Yao et al., 2011; Kaur et al., 2019; Comai et al., 2020).

The gut microbiota was a factor in intestinal inflammation of inflammatory bowel disease (Shim, 2013; Imhann et al., 2018), and the characteristics of the gut microbiota changed when intestinal inflammation occurred. In our study, Trp changed the relative abundance of four genera in LPS challenge. For example, Trp supplementation to the LPS-challenged pigs increased the abundance of *Anaerostipes*, and decreased the abundances of *Corynebacterium* and *unclassified_c__Bacteroidia* at the genus level. *Anaerostipes* converse the anaerobic inositol stereoisomers to propionate and acetate, which is crucial for adapting the change of gut nutritional supplementation (Bui et al., 2021). This is in agreement with our results that Trp supplementation to the LPS-challenged pigs increased the relative abundance of *Anaerostipes*. SCFAs have anti-inflammatory effects on the intestine (Gonçalves et al., 2018). In this study, LPS-challenge increased the concentrations of inflammatory cytokines, and Trp supplementation to the LPS-challenged pigs inhibited the increase in the concentrations of inflammatory cytokines caused by LPS. As a result, our data implied that Trp helped in maintaining the colonic mucosal microbiota homeostasis in LPS-challenged piglets by supporting beneficial bacteria colonization.

Trp metabolite acts as an endogenous ligand of AhR activation. Kynurenine and indole, the metabolites of Trp, bind and activate the AhR and its downstream effector molecules (*CYP1A1* and *CYP1B1*) in regulating intestinal immunity (Marsland, 2016). In this study, Trp supplementation to the LPS-challenged pigs increased the gene expression of *AhR*, *CYP1A1*, and *CYP1B1*. This result is consistent with a previous study finding that bacterial metabolites of Trp activated *AhR* and the downstream *CYP1A1* and *CYP1B1* gene to achieve the effect of regulating intestinal immunity and homeostasis (Zelante et al., 2013). Moreover, the activation of the AhR signaling is crucial for anti-inflammatory responses (Wenzel et al., 2020). This is in agreement with this study that the intestinal mRNA levels of *IL-1*β, *IL-6*, *IL-8*, and *TNF*-α were decreased in LPS-Trp piglets compared with the CON-LPS group. This is also consistent with this study that propionate, butyrate, and isovalerate concentrations were negatively correlated with the mRNA expression of *IL-1*β, *IL-6*, and *TNF*-α. Moreover, this result is in agreement with the previous report that SCFAs regulates cytokine and immune cell functions, which is crucial in alleviating inflammation (Louis et al., 2014; Parada Venegas et al., 2019).

Inflammation causes oxidative stress. In this study, LPS increased the oxidative stress of the colon by inhibiting the activities of antioxidant enzymes T-SOD, GSH-Px, and CAT in the gut; this finding is in agreement with the previous result (Tian et al., 2018). We found that 0.2% of Trp reduced the oxidative stresses caused by LPS in weaned pigs, which was consistent with the previous finding that Trp had antioxidant capacity (Oxenkrug, 2011). In addition, acetate, propionate, butyrate, and isovalerate concentrations were negatively correlated with MDA content; this result is also in agreement with a previous report that SCFAs, especially acetate and butyrate, reduce oxidative stress caused by high glucose and LPS in mesangial cells (Huang et al., 2017). Therefore, these results suggested that Trp alleviates oxidative stress of LPS-challenged piglets.

Inflammatory cytokines disrupts intestinal barrier function by rearranging tight junction proteins (Vancamelbeke and Vermeire, 2017). The intestinal tight junction is the major component of the intestinal epithelium’s physical barrier and governs the intestinal epithelium’s selective permeability (Pearce et al., 2018). External stressors make intestinal tight junctions vulnerable, thereby resulting in local or systemic inflammation (Gao et al., 2021). In our study, relative to the LPS-CON pigs, Trp supplementation had higher protein expression of claudin-1, occludin, and ZO-1. This study showed that concentration of acetate, propionate, and butyrate concentrations were positively correlated with *occludin* gene expression. This is also consistent with the study that SCFAs affects the gut barrier function (Li et al., 2020). Therefore, Trp contributes to the colonic epithelial barrier function in LPS-challenged piglets.

## Conclusion

These results suggest that Trp enhances intestinal health, in part, by modulating intestinal microbiota composition, improving the SCFAs, reducing inflammation, increasing antioxidant capacity, and improving intestinal barrier function.

## Data Availability Statement

The datasets presented in this study can be found in online repositories. The names of the repository/repositories and accession number(s) can be found below: https://www.ncbi.nlm.nih.gov/, SRP321842.

## Ethics Statement

The animal study was reviewed and approved by Sichuan Agricultural University Animal Care and Use Committee (SICAU-2021-0830). Written informed consent was obtained from the owners for the participation of their animals in this study.

## Author Contributions

GL, JL, and WS performed the research and analyzed the data. GJ, HZ, XC, IK, RZ, and JW contributed to the analysis and manuscript preparation. All authors contributed to the article and approved the submitted version.

## Conflict of Interest

The authors declare that the research was conducted in the absence of any commercial or financial relationships that could be construed as a potential conflict of interest.

## Publisher’s Note

All claims expressed in this article are solely those of the authors and do not necessarily represent those of their affiliated organizations, or those of the publisher, the editors and the reviewers. Any product that may be evaluated in this article, or claim that may be made by its manufacturer, is not guaranteed or endorsed by the publisher.

## Footnotes

1
http://www.drive5.com/uparse/

2
https://www.arb-silva.de

3
https://rdp.cme.msu.edu/

4
https://www.mothur.org/wiki/Download_mothur

5
http://qiime.org/install/index.html
