# Supplementary material for: Tryptophan Supplementation Enhances Intestinal Health by Improving Gut Barrier Function, Alleviating Inflammation, and Modulating Intestinal Microbiome in Lipopolysaccharide-Challenged Piglets
Source: Front Microbiol. 2022 Jul 4;13:919431. doi: 10.3389/fmicb.2022.919431 (PMC9289565; doi:10.3389/fmicb.2022.919431)
Supplement: Supplementary file 1 [file Table_1.DOCX]

**Tryptophan Supplementation Enhances Intestinal Health by Improving Gut Barrier Function, Alleviating Inflammation, and Modulating Intestinal Microbiome in Lipopolysaccharide-Challenged Piglets**

***Guangmang Liu^1*^, Jiajia Lu^1^, Weixiao Sun^1^, Gang Jia^1^, Hua Zhao^1^, Xiaoling Chen^1^, Gang Tian******^1^, Jingyi Cai^1^, In Ho Kim^2^, Ruinan Zhang^1*^ and Jing Wang^3^***

*^1^ Institute of Animal Nutrition, Key Laboratory for Animal Disease-Resistance Nutrition, Ministry of Education, Ministry of Agriculture and Rural Affairs, Key Laboratory of Sichuan province, Sichuan Agricultural University, Chengdu, Sichuan, China, ^2^ Department of Animal Resource and Science, Dankook University, Cheonan, South Korea, ^3^ Maize Research Institute, Sichuan Agricultural University, Chengdu, Sichuan, China*

**TABLE S1 |** Primer sequences used for real-time PCR

| Target Genes | Forward primer | Reverse primer | Accession number | Temperature (℃) | Product size (bp) |
| --- | --- | --- | --- | --- | --- |
| IL-1β | AAGGCCGCCAAGATATAACTGA | GCCCTCTGGGTATGGCTTTC | NM_001302388.1 | 58 | 71 |
| IL-6 | ATGCTTCCAATCTGGGTTCAA | CACAAGACCGGTGGTGATTCT | AF518322.1 | 58 | 61 |
| IL-8 | ACATCCATGAGGAAGACAGTTTGA | CGGGAACTCCACGCTAGATTC | AB057440.1 | 58 | 70 |
| TNF-α | CGACTCAGTGCCGAGATCAA | GACCTGCCCAGATTCAGCAA | JF831365.1 | 58 | 60 |
| AhR | CGTGCCAGCCGGACTCT | TTGCTGGGCTGTACTGCATCT | XM_001927795.5 | 58 | 57 |
| CYP1A1 | CATCCGGGACATCACAGACA | GCATTCTCGTCCATCCTCTTGT | NM_214412.1 | 58 | 63 |
| CYP1B1 | TAACCAGTGGTCTGTGAATCATGA | AGGAATCGGGCTGGATCAA | MF420351.1 | 58 | 68 |
| claudin-1 | GCTCCTGCCCCCGAAA | AAGGCGAAGGTTTTGGATAGG | NM_001244539.1 | 58 | 63 |
| occludin | CCTCAGGCAGCCTCATTACAG | GGGAGCCCGTTTTGAAGAC | NM_001163647.2 | 58 | 61 |
| ZO-1 | CCCAACCTCACAAATAGAAAGTGA | GCGAATAATGCCAGAGCTACGT | XM_013993251.1 | 58 | 70 |
| β-actin | TGCGGGACATCAAGGAGAA | GCCATCTCCTGCTCGAAGTC | DQ452569.1 | 58 | 59 |
